# Underestimated risks of recurrent long-range ash dispersal from northern Pacific Arc volcanoes

**DOI:** 10.1038/srep29837

**Published:** 2016-07-21

**Authors:** A. J. Bourne, P. M. Abbott, P. G. Albert, E. Cook, N. J. G. Pearce, V. Ponomareva, A. Svensson, S. M. Davies

**Affiliations:** 1 Department of Geography, College of Science, Swansea University, Swansea, UK; 2 Centre for Ice and Climate, Niels Bohr Institute, University of Copenhagen, Denmark; 3 Department of Geography and Earth Sciences, Aberystwyth University, Aberystwyth, UK; 4 Inst. of Volcanology and Seismology, Petropavlovsk-Kamchatsky, Russia

## Abstract

Widespread ash dispersal poses a significant natural hazard to society, particularly in relation to disruption to aviation. Assessing the extent of the threat of far-travelled ash clouds on flight paths is substantially hindered by an incomplete volcanic history and an underestimation of the potential reach of distant eruptive centres. The risk of extensive ash clouds to aviation is thus poorly quantified. New evidence is presented of explosive Late Pleistocene eruptions in the Pacific Arc, currently undocumented in the proximal geological record, which dispersed ash up to 8000 km from source. Twelve microscopic ash deposits or cryptotephra, invisible to the naked eye, discovered within Greenland ice-cores, and ranging in age between 11.1 and 83.7 ka b2k, are compositionally matched to northern Pacific Arc sources including Japan, Kamchatka, Cascades and Alaska. Only two cryptotephra deposits are correlated to known high-magnitude eruptions (Towada-H, Japan, *ca* 15 ka BP and Mount St Helens Set M, *ca* 28 ka BP). For the remaining 10 deposits, there is no evidence of age- and compositionally-equivalent eruptive events in regional volcanic stratigraphies. This highlights the inherent problem of under-reporting eruptions and the dangers of underestimating the long-term risk of widespread ash dispersal for trans-Pacific and trans-Atlantic flight routes.

Predicting the hazards and risk to society of widespread volcanic ash dispersal is compromised by the lack of a complete record of global volcanism with well-constrained evidence of eruptive magnitude, tephra fall volume and dispersal extent^1^,^2^. Extensive ash dispersal is recognised as the foremost volcanic hazard that can disrupt a substantial proportion of the population^3^. The risks to aviation are particularly significant, as exemplified by recent narrowly avoided air accidents (e.g. Redoubt 1989)^4^ and the substantial economic losses (US$5 billion) suffered due to the enforced airspace closure during the Eyjafjallajökull 2010 eruption^5^. Assessing the extent of the threat from future events depends entirely on a thorough understanding and compilation of volcanic histories and ash-fall distribution patterns^6^,^7^. Given the fragmentary and poorly preserved nature of the geological records, global volcanic databases^6^,^8^ are inherently affected by the under-reporting of events and spatial and temporal biases^2^,^9^. Some volcanoes are very poorly studied and under-reporting of events becomes more apparent as one goes back in time and is particularly notable for medium-scale events^2^. Recent findings and observations have highlighted that even moderate-sized events (such as the White River Ash)^10^ with short-recurrence intervals can distribute ash over much wider geographical areas than previously anticipated^10^ (Fig. 1a). Low frequency but high-magnitude events are also under-represented in the geological record and a focus on the short and most recent Holocene period may well bias any risk analysis^11^. Assessing the ash-related risk to the aviation industry with an incomplete history is thus compromised.

We present new evidence of repeated explosive Late Pleistocene eruptions in the Pacific Arc, that are hitherto undocumented in the proximal geological record and dispersed ash up to 8000 km distance from volcanic source.

## Results

**Figure 1:**
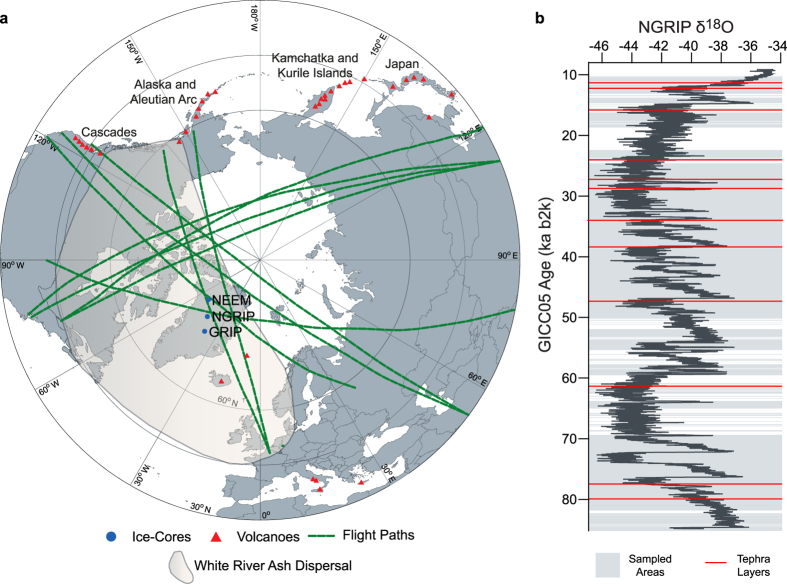
(**a**) Map showing the location of Greenland deep ice-cores investigated in this study and the main Northern Hemisphere volcanic centres active during the Quaternary. In addition, the distribution of the widespread White River Ash tephra deposit^10^ is shown as an example of a moderate-sized eruption with widespread tephra deposition (see text in main paper), along with major cross polar flight routes^36^. Map was modified from a base map in the public domain^37^. (**b**) Stratigraphic position of the 12 far-travelled cryptotephra deposits (red lines) against the NGRIP δ^18^O record^38^. Areas of the ice-cores sampled for cryptotephra content are shown in grey.

**Figure 2:**
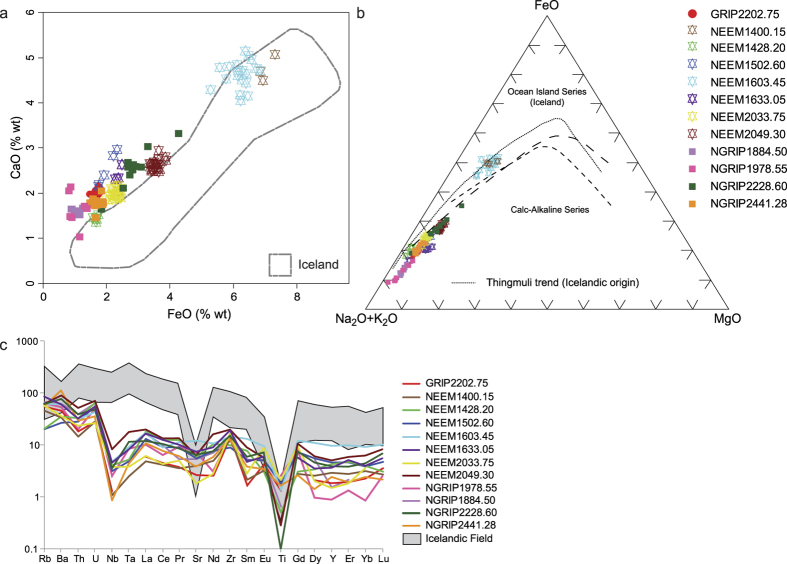
(**a**) FeO vs. CaO biplot showing the compositions of glass-shard analyses from the 12 ice-core deposits in relation to the Icelandic volcanic field (comprised of glass analyses of dacitic and rhyolitic tephra deposits from Iceland stored in the RESET database)^39^. (**b**) AFM ternary diagram showing the magma series and volcanic environment affinities of the cryptotephra deposits analysed in this study. The dashed lines show the various divisions between the calc-alkaline and the tholeiitic fields. The Thingmuli trend would be consistent with Icelandic origin whereas the ice-core cryptotephra deposits exhibit calc-alkaline composition (adapted from)^14^. (**c**) Primitive Mantle-normalised^40^ trace element profile for the ice-core cryptotephra deposits compared to tephras originating from the Icelandic field^41^. All data are normalised.

A search of the Greenland ice-cores has revealed volcanic glass particles that can be traced back to distant eruptive centres in the northern Pacific. Although, the ash-fall record preserved within the ice is swamped by recurrent local Icelandic basaltic eruptions^12^, twelve cryptotephra deposits (glass-shard concentrations insufficiently numerous to be visible to the naked eye), ranging in age between 11.1 and 83.7 ka b2k, reveal major and trace-element signatures that are inconsistent with an Icelandic anorogenic magmatic setting (Figs 1b and 2, Table 1). These cryptotephra deposits are only able to be detected by melting the ice and exploring the residual particulate material with light microscopy. The cryptotephra deposits are present throughout the last glacial period ranging in stratigraphic position from GI-21e to GS-1/Holocene transition (Fig. 1b) and are deposited during stadial and interstadial events. The glass-shard concentrations range over two orders of magnitude, with half the deposits yielding more than 95 shards, and the other half containing fewer than 36 (Table 1). This overall range in shard abundance, along with the small size of the particles (average 15–41.2 μm) is similar to the abundance and size of shards for tephras from Icelandic sources preserved in the Greenland ice (Table 1)^12^. We obtained geochemical data for each cryptotephra deposit from single-grain analyses by electron microprobe and laser ablation-inductively coupled plasma-mass spectrometry (LA-ICP-MS) (Table 1). Recent technical advances allow small individual glass shards extracted from ice-core material to be analysed for their trace element signatures^13^. Given the nature and availability of geochemical datasets for proximal deposits, both major and trace elements are used for determining tectonic setting whilst only the major elements (together with chronostratigraphic data) are used for assessing correlations to specific eruptions.

### Volcanic Setting

With the exception of NEEM 1400.15 m and NEEM 1603.45 m (sample nomenclature is defined in the methods), all deposits reveal high CaO relative to FeOt values, which distinguishes them from Icelandic eruptives of dacitic and rhyolitic composition (Fig. 2a). The same ten deposits are also calc-alkaline in composition and do not fall on the Thingmuli trend (Fig. 2b) as expected of Icelandic tephras^14^. Mantle-normalised trace-element profiles for all deposits are characterised by enrichments in the large ion lithophile elements (LILE e.g. Rb, Ba) relative to the high field strength elements (HFSE e.g. Nb, Ta, Ti) and the rare-earth elements (REE La to Lu)^15^. This feature, along with pronounced depletions in Nb, Ta and Ti, provides diagnostic fingerprints of ash generated in a subduction-tectonic setting and contrasts markedly from expected profiles for anorogenic-derived Icelandic volcanism (Fig. 2c)^15^. Despite showing major element affinity to Icelandic sources, trace-element data for NEEM 1400.15 m and NEEM 1603.45 m also point towards a subduction-tectonic setting. The closest active subduction zone settings to Greenland are the northern Pacific volcanic arcs and the compositional signatures reported here are consistent with those of the products from the Cascadian, Alaska-Aleutian, Kamchatka-Kurile and Japanese arcs (Fig. 1). The prevailing wind direction and abundant East Asian dust transport to Greenland during the last glacial period support this tephra transport pathway^16^.

### Correlation of Cryptotephra Deposits

**Figure 3:**
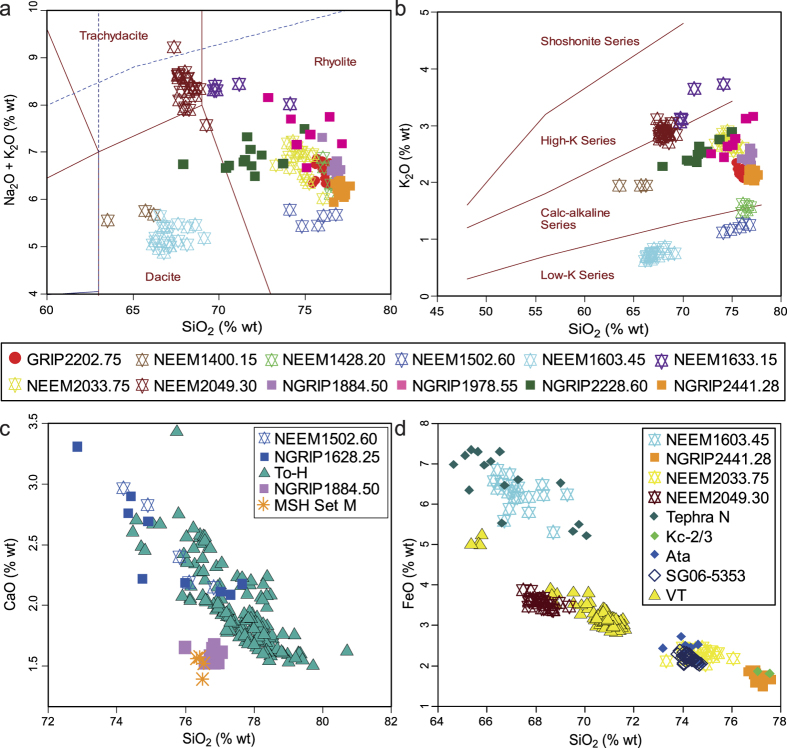
(**a**) Total alkali vs. silica diagram^42^. (**b**) SiO_2_-K_2_O plot^43^. (**c**) Comparison of glass shard analyses from samples NEEM 1502.60 m/NGRIP1628.25 m with those for glass of the Towada-H^46^ deposits from Honshu, Japan, and NGRIP 1884.50 m with Mount St. Helens Set M^47^. (**d**) Comparison of glass shard analyses from the ice-core cryptotephra deposits with those of potential volcanic sources, Kurile Islands (Tephra N), Kutcharo caldera (Kc-2/3)^46^, Ata caldera (Ata and SG06-5353)^20^ and the Eastern Aleutian Arc (VT)^48^. All data have been normalised.

Glass shards from the cryptotephra deposits range from dacitic in composition with SiO_2_ values of 63.52–66.22 wt% (NEEM 1400.15 m) to rhyolitic with SiO_2_ values of 76.68–77.61 wt% (NGRIP 2441.28 m) (Table 1, Fig. 3a). The potassium values of the twelve deposits also show significant variation, with NEEM 1603.45 m (K_2_O = 0.62–0.86 wt%) and NEEM 1502.60 m (K_2_O = 1.12–1.28 wt%) classified in the Low-K series and NEEM 1633.15 m (K_2_O = 3.05–3.73 wt%) and NEEM 2049.30 m (K_2_O = 2.70–3.11 wt%) classified in the High-K series with the remaining eight deposits classified in the calc-alkaline series (Table 1, Fig. 3b).

Although the compositional data point towards northern Pacific Arc sources, matching the ash deposits to reported eruptions of similar age and composition is difficult and could be compounded by differences in compositional ranges between typically more heterogeneous proximal deposits and the more restricted ranges of distal deposits^17^,^18^. We draw on the eruptive data compiled in the database of large magnitude explosive volcanic eruptions (LaMEVE)^6^ and are only able to find two potential matches for our ice-core cryptotephra deposits. The major element signature for NEEM 1502.60 m (which is correlated to NGRIP 1628.25 m)^19^ overlaps with that for the Towada-H eruption, associated with a magnitude 6.9, VEI 6 event on north-eastern Honshu Island, Japan (Fig. 3c). The NEEM age of 15.7 ± 0.2 ka b2k is consistent with the proximal age of this deposit (10.19–18.55 cal ka BP)^20^. Geochemical and age similarities are also observed between NGRIP 1884.50 m (28.8 ± 0.8 ka b2k) and the Mount St Helens Set M complex (magnitude 5.3, VEI 5) constrained in age to between 28 and 18 ka BP^21^ (Fig. 3c).

Where possible, potential sources are suggested for the remainder of the tephras (Table 1), but no correlations can be made to specific eruptions reported in the literature due to age discrepancies, in some cases up to 30,000 years. Some of our suggested sources based on comparisons of available major element data include the Ata and Kutcharo (Japan) calderas to NEEM 2033.75 m and NGRIP 2441.28 m, respectively, but the ice-core ages for these events are mismatched with the local eruption stratigraphy and the proposed ages for the formation of these calderas (Fig. 3d). We argue therefore that these could represent new, previously undocumented eruptions from these sources but we do not rule out other more distal Pacific rim sources such as Indonesia and the Philippines. However, no compositional matches of similar age are identified^22^.

### Chemical Records of Volcanism

Although the discovery of these cryptotephra deposits demonstrates the significant dispersal of ash from non-Icelandic eruptions, only three of these deposits are marked by any concurrent volcanic aerosol deposition preserved in polar ice-cores, in these cases represented as chemical matchpoints between ice-cores^23^. The remaining nine ash deposits have been dispersed over 8000 km to Greenland but have no associated volcanic aerosol signal preserved in the ice. All of these nine cryptotephra horizons were deposited during cold stadial events (Table 1), which are characterised by high background levels of sulphate deposition and suppressed Electrical Conductivity Measurements (ECM) and Di-electrical Profiling (DEP) levels due to alkaline dust deposition. Therefore, volcanic aerosol indicators for these eruptions may be hidden or have been subdued because of the timing of deposition. Long and unbroken records of ice acidity and sulphate aerosol deposition preserved within the ice typically provide the focus for global volcanism indices^24^. Our results thus demonstrate that many significant and widespread eruptives will be overlooked during the glacial period if the focus remains solely on aerosol deposition.

## Discussion

The discovery of several far-travelled volcanic ash clouds highlight the inherent problem of under-reporting eruptions and the under-representation of events from volcanoes with long repose times^2^,^9^. It is clear that many more eruptions in the past have deposited ash over much larger geographical areas than previously anticipated. Given that our ice-core data-set is in a distal location and with only partial matches to near-source volcanic deposits, the magnitude and explosivity of the eruptive events that generated the ash clouds are unknown. We do not believe that ash dispersal is enhanced by intensified atmospheric circulation during stadial periods^25^ because some of the tephras are also deposited during interstadial periods (Fig. 1b). However, we argue that the dacitic to rhyolitic chemical compositions and the extent of ash dispersal point towards explosive and voluminous eruptions that characterise these Si-rich (acidic) magmas^26^. We postulate that they are probably of medium magnitude as these are the ones most likely to be under-reported and undocumented^2^. However, it is also conceivable that these are large-magnitude events with long return intervals that have been missed in the geological records due to ice removal of proximal deposits, for example. Recent mathematical and modelling analysis of the Japanese eruptions reported in the LaMEVE database^6^ suggests that a high percentage of high-magnitude eruptions are under-reported over the full Quaternary period^27^. Whilst not all of the Greenland ice-cores between 11 and 85 ka has been sampled (Fig. 1b), the recurrent interval is approximately every 6000 years over the whole period and every 3000 years if only the 10–30 ka period is considered. Volcanic risk assessments are frequently based on the Holocene period due to the more complete nature of the record^3^,^11^, but our results from the last glacial reinforce the urgency to consider longer-term assessments and extra-regional spatial scales to fully capture the impact of these far-reaching ash clouds. Based solely on Holocene data, Kamchatka and Japan are two of the volcanic systems in the Asian-Pacific region showing increased volcanic ash hazards at longer repose periods^11^.

During the last 100,000 years the most widespread ash deposits include the Younger Toba Tuff (YTT) from Sumatra, Indonesia, found over 7000 km west of the source volcano in Lake Malawi (magnitude 8.8, VEI 8)^6^,^28^, the White River Ash dispersed approximately 7000 km east from Mt Churchill, Alaska (magnitude 6.1, VEI 6)^6^,^10^ (Fig. 1a), the Vedde Ash dispersed from the Katla Volcano in Iceland to Slovenia and western Russia (magnitude 5.8, VEI 6)^6^,^29^ and ash from the Campanian Ignimbrite (CI) eruption of Campi Flegrei, Italy, was dispersed more than 2200 km east to Russia and over 1000 km southward to the north African coast (magnitude 7.1, VEI 7)^6^,^30^ (Fig. 1a). Whilst two of these far-travelled eruptions, the YTT and CI, are both classified as super-eruptions, the White River Ash and Vedde Ash are one to two orders of magnitude lower volume in comparison. In addition, the Pacific Arc tephras identified in Greenland, do not correlate with some of the most explosive and high-magnitude eruptions from this region (e.g. the Japanese VEI 8 eruptions, AT and Aso-4 eruptions)^6^,^20^. Our discoveries of potential gaps in the Japanese volcanic stratigraphy support previous findings^27^ and highlight the importance of distal tephra studies for building complete eruptive histories. These eruptions were probably of medium magnitude, capable of generating widespread ash clouds, but we cannot rule out the possibility that these are as yet undiscovered large-magnitude eruptions^27^. Such far-travelled ash clouds are reported to have caused damage to aircraft in the past^31^. Ash probability maps based on historical eruptions in the North Pacific do not predict ash leaving the region^32^. The risk of disruption to trans-Pacific and trans-Atlantic flight routes by recurrent ash dispersal from the Pacific Arc is therefore greater than previously thought.

## Methods

The 12 deposits presented here have been identified as part of continued efforts to establish a tephrochronological framework for the Greenland ice-cores^12^. Long and continuous time-intervals have been sampled throughout the last glacial period rather than targeted sampling intervals dictated by the ages of known high-magnitude eruptions. The ice-core samples were processed for tephra investigations according to the procedures outlined in^12^. Cryptotephra deposits identified in the ice have been given unique labels based on the name of the ice-core and the basal depth of the sample containing the ash-sized glass shards e.g. the cryptotephra found in NGRIP sample 1399.95–1400.15 m will be NGRIP 1400.15 m.

Both major and trace elements were used to determine the tectonic setting of the volcanoes that generated the tephra deposits found in the cores. Identification of potential correlative tephra deposits was undertaken by compiling a list of dacitic and rhyolitic eruptions from northern Pacific Arc volcanoes with a VEI of ≥5 aged between 10 and 80 ka BP from the LaMEVE database^6^. Due to the limited availability of proximal geochemical datasets, only major element data are used for the attempted correlation to specific eruptions.

Major element results on single-glass shards were obtained by electron-probe microanalysis (EPMA) during seven analytical periods at the Tephra Analytical Unit at the University of Edinburgh, UK. A Cameca SX-100 electron microprobe with five vertical wavelength dispersive spectrometers was employed to analyse 10 major and minor elements within individual glass shards. Both 3- and 5- μm beam diameters were used, according to the grain-size of the glass shards making up the samples, and the operating conditions followed those outlined in^33^. Secondary standard analyses of Lipari obsidian and BCR-2 G basalt were analysed at the beginning and end of each day, as well as at regular intervals between analyses of samples to monitor the accuracy and precision of the instrument and data. The major oxide data are normalised to an anhydrous basis and raw geochemical results, including the operating conditions, beam diameter employed for each sample, and standard data are provided in the Supplementary information.

LA-ICP-MS analyses were performed at Aberystwyth University to analyse the trace element composition of single glass shards from each deposit. A Coherent GeoLas 193 nm Excimer laser system was coupled to a Thermo Finnegan Element 2 high-resolution sector field ICP-MS^13^. Analyses were performed with a 10 μm laser beam diameter, the laser was pulsed at 5 Hz with a fluence of 10 J/cm^2^, and had a flash duration of ~20 ns. Each acquisition took 24 s, with Ar as the carrier gas. The internal standard used was ^29^Si (determined by EPMA analysis). Geochemically distinct MPI-DING^34^ reference glasses were used to monitor the analytical accuracy and can be found in the Supplementary data. Analyses using a 10 μm laser beam diameter are at the limit of instrumental sensitivity for many elements^13^, and while analytical accuracy remains consistent between sessions, slight variations in operating conditions (instrument sensitivity, tuning, analytical blanks) can lead to differences in signal/noise, (and thus detection limits) and analytical precision. Given the small number of glass-shard analyses available from these cryptotephras deposits, these methodological challenges should be considered if these data are utilised in future comparisons.

Ages are assigned to each tephra according to the NGRIP GICC05 annual-layer counted chronology that has also been transferred to all deep ice-cores in Greenland^23^,^35^. This timescale delimits age errors based on the concept of maximum counting errors (MCE), which are considered to represent 2σ uncertainties^35^.

## Additional Information

**How to cite this article**: Bourne, A. J. *et al*. Underestimated risks of recurrent long-range ash dispersal from northern Pacific Arc volcanoes. *Sci. Rep.* **6**, 29837; doi: 10.1038/srep29837 (2016).

## Supplementary Material

Supplementary Information

## Figures and Tables

**Table 1:** Northern Pacific cryptotephra deposits present in the Greenland ice-core.

Tephra label	Depth Range	Shards	Period	Age	MCE	Ave. grain size (μm)	Max grain size (μm)	Min grain size (μm)	Total Alkali vs. SiO_2_ Classification^42^	SiO_2_ vs. K_2_O Classification^43^	Potential Volcanic Source
NEEM 1400.15 m	1399.95–1400.15	23	GI-1	11202	95	28.0	45.0	20.0	Dacite	Calc-alkaline	Unknown
NEEM 1428.20 m	1428.00–1428.20	15	GS-1	12127	113	29.5	52.5	15.0	Rhyolite	Calc-alkaline /Low-K	Unknown
NEEM 1502.60 m	1502.45–1502.60	22	GS-2.1a	15706	226	29.6	50.0	15.0	Rhyolite	Low-K	Towada, Japan
NEEM 1603.45 m	1603.25–1603.45	113	GS-3	23960	634	24.0	40.0	12.5	Dacite	Low-K	Kurile Islands
NEEM 1633.05 m	1632.95–1633.05	36	GS-3	27171	800	15.0	50.0	10.0	Rhyolite	High-K	Unknown
NGRIP 1884.50 m	1884.30–1884.50	5	GI-4	28800	894	23.5	75.0	12.5	Rhyolite	Calc-alkaline	Mt St Helens, Cascades
NGRIP 1978.55 m	1978.35–1978.55	152	GS-7	33980	1232	32.1	47.5	22.5	Rhyolite	Calc-alkaline	Unknown
GRIP 2202.75 m	2202.60–2202.75	638	GS-9	38371	1456	41.2	62.5	27.5	Rhyolite	Calc-alkaline	Unknown
NGRIP 2228.60 m	2228.40–2228.60	461	GS-13	47320	1938	30.3	52.5	17.5	Dacite/Rhyolite	Calc-alkaline	Unknown
NGRIP 2441.28 m	2441.24–2441.28	24	GS-19.1	61425	2675	38.0	50.0	30.0	Rhyolite	Calc-alkaline	Kutcharo, Japan
NEEM 2033.75 m	2033.55–2033.75	95	GS-21.1	77559	3450	22.5	32.5	12.5	Rhyolite	Calc-alkaline	Ata Caldera, Japan
NEEM 2049.30 m	2049.15–2049.30	232	GI-21.1e	80065	3600	39.1	72.5	17.5	Trachydacite	High-K	Eastern Aleutian Arc

For each cryptotephra the following information is provided: depth interval of ice sampled, shard numbers identified per sample, climatic event within which tephra was deposited^23^, age, grain-size information, geochemical composition, and likely volcanic source. Shard numbers are given for each sample but are not directly comparable with one another due to differences in sample volume. The climatic events are defined based on the event stratigraphy presented in^23^. Ages are in b2k (before 2000 CE) and represent the age of the basal depth of the ice sample containing the glass shards. The ages are obtained from the GICC05 timescale in steps of 20 years for the NGRIP core^35^ and the GRIP core^44^, and in steps of 0.55 cm for the NEEM core^45^. MCE = maximum counting error; in a standard deviation context, the maximum counting error should be regarded as 2 sigma^35^.
